# Can We Determine Osteoarthritis Severity Based on Systemic Immuno-Inflammatory Index?

**DOI:** 10.3390/diagnostics15121556

**Published:** 2025-06-18

**Authors:** Bilge Kagan Yilmaz, Recep Altin, Alper Sari

**Affiliations:** 1 Department of Orthopaedic and Traumatology, Afyonkarahisar Health Science University, 03030 Afyonkarahisar, Turkey; recep.altin@afsu.edu.tr; 2 Department of Internal Medicine, Afyonkarahisar Health Science University, 03030 Afyonkarahisar, Turkey; alpersari_@hotmail.com

**Keywords:** systemic immune-inflammatory index, osteoarthritis, inflammatory, degenerative

## Abstract

**Background:** Osteoarthritis (OA) is one of the common joint diseases. Hematologic markers have been investigated to determine its severity and predict the prognosis of joint diseases. In this study, we investigated whether the systemic immune-inflammatory index (SII) is a marker for assessing the severity of OA. **Methods**: The records of patients diagnosed with OA at various stages between 1 January 2020 and 1 January 2022 were retrospectively analyzed. Patients aged 18–75 years with complete blood count within the last 15 days and not taking anti-inflammatory drugs were included in the study. Patients were classified according to the Kellgren–Lawrance classification as stage 1-2-3 mild to moderate OA (Group I) and stage 4 severe OA (Group II). A total of 1580 patients were diagnosed with knee OA and 946 were included in the study. Of the patients, 246 (26%) were male and 700 (74%) were female. The mean age of the patients was 61.00 (53.00–68.00) years. **Results**: There were 449 (47.5%) patients in Group I and 497 (52.5%) patients in Group II. Statistically significant differences were found between the groups in age, gender, hemoglobin, lymphocytes, and SII (*p* < 0.05). An SII value of 627.9 was found to distinguish severe OA from mild–moderate OA with 42.5% sensitivity and 70.6% specificity. **Conclusions**: Although this study is the first in the literature, it shows that SII has limited predictive value in assessing the severity of knee OA. Future research should focus on longitudinal studies to establish causality and explore therapeutic implications.

## 1. Introduction

Osteoarthritis (OA) is the most common joint disease worldwide, with its prevalence increasing due to the aging population and overall population growth [1,2]. Traditionally, OA is defined as a degenerative joint disease characterized by synovial inflammation, subchondral bone remodeling, and subsequent cartilage degradation [3,4]. The disease is characterized by progressive cartilage degradation, joint space narrowing, and remodeling of adjacent bone. It is regarded as a complex disease whose pathogenesis is not completely understood. Recent studies have highlighted the inflammatory aspects of OA, demonstrating that inflammation is a critical factor associated with the progression of cartilage destruction and clinical manifestations such as joint pain, swelling, synovial fluid effusion, and synovitis [5,6]. Synovial inflammation, which can occur in the early stages of OA, leads to synovitis. This condition is associated with alterations in proinflammatory cytokines and adipokines, which are implicated in OA progression [7,8,9]. These processes collectively lead to degenerative changes in cartilage and the progression of OA symptoms. Once considered a cartilage-specific condition, OA is now understood as a multifaceted disease involving inflammatory mediators originating from cartilage, bone, and synovium [10].

Various markers such as tumor necrosis factor-α (TNF-α), C-reactive protein (CRP), and interleukin-6 (IL-6) have been used to determine the severity and prognosis of OA [11,12]. However, their costs of measuring are high. On the other hand, complete blood count (CBC)-derived inflammatory markers have emerged as potential indicators of systemic inflammation due to their accessibility and affordability. One of these markers, the neutrophil–lymphocyte ratio (NLR), is based on increased neutrophil numbers and decreased lymphocyte numbers during stress responses and inflammation and is currently being evaluated in studies of rheumatologic diseases and malignancies [13]. Platelet-to-lymphocyte ratio (PLR) is a marker associated with platelet aggregation and systemic inflammation, thus allowing the assessment of inflammatory coagulation reactions and platelet activation resulting from the systemic inflammatory response [14,15,16]. A more recent parameter, the systemic immune-inflammation index (SII), combines lymphocyte, neutrophil, and platelet counts into a single marker. The SII is easily calculated by multiplying the neutrophil and platelet counts and then dividing the result by the lymphocyte count. SII has shown potential as a prognostic indicator in various conditions, including malignant tumors [17,18], coronary artery disease [19], acute ischemic stroke [20], and premature rupture of membranes [21]. SII is hypothesized to provide a more comprehensive representation of systemic inflammation compared to NLR or PLR alone.

To date, no studies have explored the association between SII and disease severity in OA patients. Therefore, the present study aims to investigate the potential of SII as a prognostic marker for assessing OA severity.

## 2. Materials and Methods

### 2.1. Study Design

The present study included knee OA patients diagnosed at the orthopedics and traumatology outpatient clinic of Afyonkarahisar Health Sciences University Hospital (Afyonkarahisar, Turkey) between 1 January 2020 and 1 January 2022. The medical records of these patients were retrospectively reviewed by orthopedics and traumatology specialists. The study was conducted according to the guidelines of the Declaration of Helsinki and approved by the Ethics Committee of Afyonkarahisar Health Science University (protocol code 2023/3 and date of approval 3 March 2023).

### 2.2. Selection of Study Population

Inclusion criteria were complete roentgenograms, age range of 18–75 years, and complete blood count within the last 15 days. Exclusion criteria were age below 18 years and above 75 years, hypertension, coronary heart disease, angina pectoris, myocardial infarction, heart failure, a history of smoking, acute or chronic infectious diseases, diabetes mellitus, obesity, asthma, chronic obstructive pulmonary disease, peripheral or cerebral vascular disease, hematological disorders (such as myeloproliferative disorders, hemoglobin >16.5 g/dL, anemia, thrombocytopenia, etc.), dyslipidemia, other musculoskeletal disease (rheumatoid arthritis, etc.), autoimmune or metabolic disorders, cancers, pregnancy, abnormal liver or renal function tests, malignancies, thrombosis or chronic renal insufficiency, musculoskeletal surgery or injury within past 2 months, and anti-inflammatory drug use in the last 15 days. Demographic data (age, gender) and complete blood parameters of knee OA patients were obtained. Standard knee roentgenograms were taken from the patients in anteroposterior and lateral positions, with the patients standing and at the same distance from the device. Knee roentgenograms were radiologically staged by two different orthopedists according to the Kellgren–Lawrance score [22]. According to the Kellgren–Lawrance classification, patients with stage 1-2-3 mild to moderate OA were divided into two groups as Group I and patients with stage 4 severe OA as Group II.

A retrospective review of 1580 patients diagnosed with knee OA was performed. A total of 332 patients over 75 years of age, 17 patients with active infection, 24 patients with inflammatory rheumatologic disease diagnosis, 19 patients with active malignancy and receiving treatment, 8 patients with known autoimmune diagnosis, and 234 patients who used anti-inflammatory drugs in the last 15 days were excluded from the study. There were 946 patients who met the study criteria included in the study.

### 2.3. Data Collection

Medical records, erythrocyte sedimentation rate (ESR), C-reactive protein (CRP), white blood cell count (WBC), neutrophil-to-lymphocyte ratio (NLR), platelet count, platelet distribution width (PDW), red blood cell distribution width (RDW), rapid plasma reagin (RPR), platelet-to-lymphocyte ratio (PLR), mean platelet volume (MPV), and systemic immune-inflammation index (SII) levels were retrospectively recorded from patients’ files. For complete blood count analysis, disposable vacuum-sealed sterile tubes containing 5.40 mg of K2-ethylenediaminetetraacetic acid (K2-EDTA) were utilized. All blood samples were studied within less than one hour after sampling. The complete blood count analyses, based on technique of laser flow cytometry scattergrams, were performed in the same analyzer (Sysmex XN-2000, Kobe, Japan) in the central laboratory of our institution, which is routinely checked every day. CBC parameters of participants were recorded from the same computerized database. According to the laboratory on which the study data were based, the normal value range was 3.5–10.5 × 10^9^/L for WBC, 5–20 mm/h for ESR, 0–5 mg/L for CRP, 10–16 fL for PWD, 11.7–14.4% for RDW, and 9.4–12.4 fL for MPV.

Neutrophil, lymphocyte, platelet, and monocyte values obtained from complete blood count tests were used. The SII is calculated by multiplying the neutrophil and platelet counts, and then dividing the result by the lymphocyte count, as expressed in the following formula:

SII: (Neutrophil Count × Platelet Count)/Lymphocyte Count

### 2.4. Statistical Analysis

Statistical analyses of the study were performed using the Statistical Package for Social Sciences (SPSS) (Version 20.0, Chicago, IL, USA). Non-parametric tests were chosen due to the non-normal distribution of several continuous variables, as determined by the Kolmogorov–Smirnov test. Continuous data conforming to non-parametric distribution is given as median and inter quartile range (IQR), and categorical data as percentage (%). Descriptive statistics were used to determine the characteristics of patients and group comparisons were made by Mann–Whitney U test. Univariate and multivariate logistic regression analyses were used to determine risk factors for advanced OA. ROC analysis was used to evaluate the sensitivity and specificity of clinical parameters in terms of diagnosis. In statistical analyses, a *p* value less than 0.05 was considered significant.

## 3. Results

Of the 946 patients, 700 (74%) were female, and the median age of all patients was 60.10 (53.00–68.00) years. According to the Kellgren–Lawrence classification, 104 patients (11%) were at stage 1, 208 (22%) at stage 2, 137 (14.5%) at stage 3, and 497 (52.5%) at stage 4. A total of 449 patients (47.5%) were included in Group I, while 497 patients (52.5%) were in Group II. Statistically significant differences were found between the groups in age, gender, hemoglobin, lymphocyte, neutrophil, and SII (*p* < 0.05). There was no statistically significant difference between the groups in terms of WBC and platelet (*p* > 0.05). Laboratory and demographic data of the patients are shown in Table 1.

**Figure 1:**
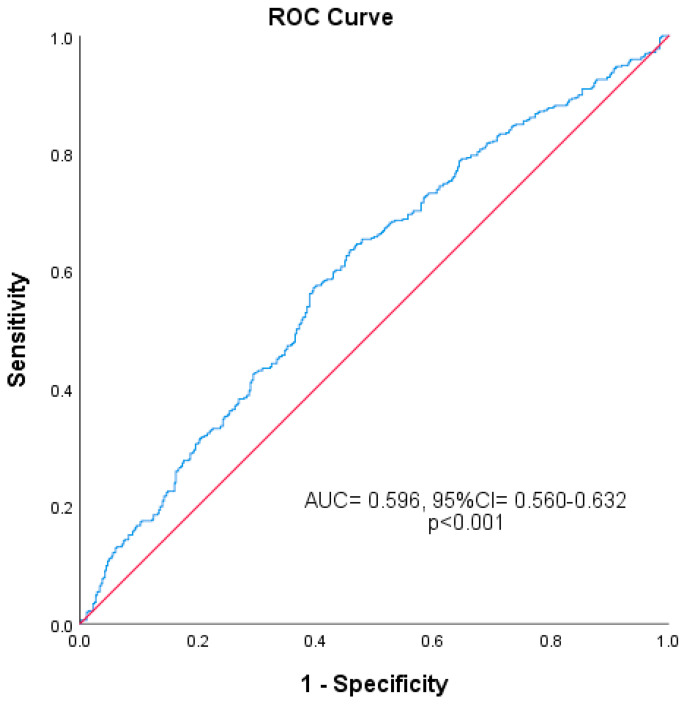
Predictive power of SII for advanced OA (AUC: 0.596; 95% CI: 0.560–0.632).

For Groups I and II, an SII value of 627.9 was found to differentiate severe OA from mild to moderate OA with a sensitivity of 42.5% and specificity of 70.6%. The plotted ROC curve is shown in Figure 1 (AUC: 0.596; 95% CI: 0.560–0.632). In ROC analysis, 0.596 was found to be well below the clinically useful level. Although this decrease in sensitivity and specificity is significant, the main model performance fit is low. When 946 patients were divided into two groups according to the threshold SII value of 627.9, 603 patients had SII values < 627.9, while 343 patients had SII values > 627.9. The severity of osteoarthritis was significantly different between these two groups (Table 2).

Univariate analysis showed that age, hemoglobin, and SII were associated with OA severity. In multivariate correlation analysis, age and SII > 627.9 were independent risk factors for OA severity (Table 3).

## 4. Discussion

The primary finding of this study is that although the systemic immune-inflammatory index (SII)—a simple ratio derived from the widely used and cost-effective complete blood count (CBC)—offers relevant insights into the radiographic severity of knee OA, its discriminatory power remains below the threshold of clinical applicability. Although SII is associated with OA severity, its use as a sole diagnostic tool is limited by its low sensitivity and specificity.

Although osteoarthritis is traditionally classified as a non-inflammatory disease, emerging evidence suggests that low-grade inflammation contributes significantly to its pathogenesis [9]. This study investigated the association between disease severity and the SII in OA patients, examining the potential of SII as a prognostic marker for assessing disease severity. The findings do not support the hypothesis, indicating that SII is not a valuable predictor for assessing OA progression. Patients with severe knee OA exhibited higher SII levels compared to those with mild to moderate OA. An SII value ≥ 627.9 predicted the severity of knee OA with a sensitivity of 42.5% and a specificity of 70.6%. However, this was not clinically significant, suggesting that SII is not useful as a marker for knee OA severity.

OA is the most common type of arthritis, affecting 27 million people in the United States alone [23]. OA is common worldwide, and the risk of OA is known to increase especially after the age of 40 years [24]. It is a painful, disabling disease that affects the synovial joints and causes a significantly reduced quality of life. The exact etiology of OA is still relatively unknown, but it is known that many risk factors are involved, such as age, genetic predisposition, malalignment, obesity, and acute joint injury [25,26,27,28,29]. Approximately 50% of the population aged ≥65 years is affected by knee OA; however, in some cases, it can also affect the younger population [30]. As is consistent with the literature, we found that age is an independent predictor of knee OA. In univariate analysis, we found that age and SII were associated with OA severity. In multivariate correlation analysis, we found that age and SII > 627.9 were independent risk factors for OA severity. However, the blood SII value obtained is not useful as a marker for knee OA severity.

OA was considered as a non-inflammatory disease in the past. Recent studies suggest that low-grade inflammation is part of the pathogenesis of OA. Low-grade inflammation results in increased production of proinflammatory cytokines in synovial membranes. The localized production of inflammatory mediators plays a crucial role in promoting cartilage degradation and stimulating the activation of synovial cells. The increase in proinflammatory cytokines also contributes to pathogenesis [31]. Smith et al. reported that there was thickening and inflammatory cell infiltration in synovial membranes in all patients, regardless of the degree of OA, but the most significant changes were in the advanced OA group. They also reported that IL-1 alpha, IL-1 beta, and TNF-alpha were produced in the synovial membranes of all patients with OA. As a result, they stated that chronic inflammatory changes caused synovitis in patients with early OA with the production of proinflammatory cytokines, which may result in the production of cytokines that may contribute to the pathogenesis of OA [31]. In this study, statistically significant differences were found between the groups in lymphocytes and neutrophils. The increase in these inflammation-related hematologic parameters in patients with severe OA supports the hypothesis that inflammation plays a role in the pathogenesis of OA in accordance with the literature.

Qin et al. [13] evaluated the levels of NLR, PLR, and MPV in adult systemic lupus erythematosus (SLE) patients and investigated their clinical significance. By multiple regression analysis, they stated that NLR may be independently associated with SLE disease activity. As a result, they emphasized that NLR and PLR may reflect the inflammatory response and disease activity in SLE patients. Fu et al. [14] investigated whether NLR and PLR are effective in assessing disease activity in rheumatoid arthritis (RA). As a result, they concluded that NLR and PLR may be potential indices in assessing RA disease activity. In another study, Hira et al. [32] compared the hematologic parameters of 118 OA patients and 145 healthy individuals. They found that NLR and PLR values were significantly higher in OA patients. Taşoğlu et al. [33] compared the NLRs of 176 patients diagnosed with OA according to the Kellgren–Lawrance classification with stage 1,2,3 in the mild–moderate OA group and stage 4 in the severe OA group. They found that the NLR level was significantly higher in the severe OA group. In RoC curve analysis, they reported that NLR ≥ 2.1 had 50% sensitivity and 77% specificity in predicting severe OA. These studies support the inflammation hypothesis of OA pathogenesis. Based on this, in our study, we found that SII was significantly higher in the severe OA group and an SII value of 627.9 could distinguish severe OA from mild–moderate OA with a sensitivity of 42.5% and specificity of 70.6%. However, contrary to the literature, the clinical benefit of this value was low. Additionally, we believe that the predictive value of SII in SLE or malignancies may not be directly transferable to OA.

SII is an objective marker reflecting systemic inflammation and is easy to calculate with the neutrophil × platelet/lymphocyte count formula. The formula has been widely examined in the literature and remains a relevant tool in current research. Wu et al. [34] investigated the relationship between SII and disease activity in patients with ankylosing spondylitis (AS). In a retrospective study including 136 AS patients and 63 healthy individuals, they showed that SII increased in AS. They emphasized that SII could be a new indicator in monitoring AS disease activity. Wang et al. [35] evaluated the association between SII and all-cause mortality in older adults undergoing surgery for hip fractures. This prospective cohort study included 290 patients with a mean age of 77.6 years. Multivariate Cox analysis revealed that each 100-unit increase in SII was associated with an 8% increased hazard of death at 1-year follow-up and a 9% increased hazard of death at final follow-up. Xia et al. [36] prospectively evaluated the association between SII and all-cause mortality and cardiovascular mortality in a 20-year cohort study of 42,875 adults. They reported that adults with SII > 655.56 had higher rates of all-cause mortality and cardiovascular mortality than those with SII levels < 335.36. They also emphasized that increased SII in the general population over 60 years of age was closely associated with an increased risk of all-cause mortality. In recent years, the scope of application of the SII has been progressively expanding, with an increasing number of studies demonstrating its potential utility in predicting disease severity and monitoring treatment responses [34,35,36]. Based on these findings, we hypothesized that SII could serve as a prognostic marker for assessing the severity of OA. However, our data indicate that the discriminatory power of SII in evaluating OA severity falls below the threshold for clinical applicability. While a statistical association between elevated SII and OA severity was found, the low sensitivity and specificity values observed limit its clinical applicability. Hence, SII should not be considered a reliable standalone diagnostic tool for OA severity

SII has also begun to be widely used in patients diagnosed with malignancy. SII has been used as a predictor of mortality in patients with malignancies and high SII values have been associated with increased mortality risk [37,38,39]. He et al. [37] examined the association of SII with mortality in patients with arteriosclerotic cardiovascular disease. They found that increased SII values were associated with poor survival in individuals with arteriosclerotic cardiovascular disease in a study of 2595 adults. Huang et al. [38] compared preoperative and postoperative SII values to estimate postoperative prognosis in patients operated on for endometrial carcinoma. A total of 362 patients were included in the retrospective study. Blood routines were examined within 1 week before surgery to calculate SII, NLR, PLR, and MLR, and within 3 days after surgery to measure SII. They reported that preoperative SII was associated with age (*p* = 0.009), FIGO stage (*p* = 0.02), and menopause (*p* = 0.014), and postoperative SII was also associated with menopause (*p* = 0.014). Multivariate analysis found that lymphatic invasion and postoperative SII were independent prognostic factors of overall survival (*p* < 0.05). As a result, they emphasized that postoperative SII was an independent prognostic factor in patients with endometrial carcinoma. Tian et al. [39] evaluated 14 articles including 2721 patients in their meta-analysis. They investigated the relationship between SII and the therapeutic effect of immune checkpoint inhibitors. They proved that high SII levels are closely associated with poor prognosis in cancer patients receiving immune checkpoint inhibitors and an SII value of 750 is suitable as a cut-off value.

Biomarkers obtained from blood counts have also begun to be used in the field of orthopedics and trauma. Moldovan et al. [40] retrospectively evaluated the relationship between surgical trauma and the predictive value of inflammatory indices in 129 patients who underwent surgery for acute hip fractures aged >70 years. In the ROC curve analysis, they found that postoperative SII > 1564.74 was a more reliable predictor of surgical trauma in terms of specificity (58.1%) and sensitivity (56.7%). Yao et al. [16] investigated the relationship among NLR, PLR, and SII on the presence of postoperative pneumonia (POP) in geriatric patients with hip fractures. A total of 1199 elderly patients with hip fractures who underwent surgical treatment were included in this retrospective study. They reported that 111 (9.26%) of them developed postoperative pneumonia. They found that NLR showed the highest predictive value for POP in elderly patients with hip fractures compared to PLR and SII (AUC = 0.648, 95% CI 0.594–0.701). They reported that a high NLR was significantly associated with an increased incidence of POP using the optimal cut-off value of 5.84 (OR = 2.24, 95% CI 1.43–3.51). As a result, they found that NLR showed the highest reliability as a predictor of POP in elderly patients with hip fractures. There are studies indicating that it can also be used to evaluate the severity of fracture types. Wang et al. [41] retrospectively examined the correlation between imaging severity and NLR in blood in 223 patients with isolated tibial plateau fractures. They grouped patients with Schatzker types I–IV as mild–moderate and those with Schatzker types V–VI as severe. They found that NLR, hemoglobin, neutrophil count, and platelet count were significantly different between the two groups. They concluded that NLR level is a useful biomarker for predicting the severity of isolated tibial plateau fractures in young and middle-aged adults. Combining SII with other inflammatory markers such as CRP or NLR may improve predictive accuracy. Future studies should explore composite biomarker models for more robust OA severity assessment.

There are limited number of studies in the literature examining hematologic parameters in OA patients. There is no study examining the correlation between SII and OA severity. To the best of our knowledge, our study, the first in the literature, demonstrated that blood SII > 627.9 is an independent risk factor for severe knee OA. However, the clinical benefit of this value was found to be low.

The most important limitation of this study is that it is a retrospective study. In addition, it cannot explain the pathogenesis of high blood SII in severe knee OA. Patients with bilateral osteoarthritis and RA were not included in the study. This may affect the results because SII may have different values in advanced osteoarthritis and RA cases. In addition, patients with osteoarthritis of other joints such as hip and spine were not excluded from the study. This may have caused the SII to be falsely elevated due to the involvement of more than one joint. The presence of both conditions cannot be applied to the general OA population. The use of a single blood sample does not allow the assessment of changes in blood SII over time. Furthermore, larger patient groups representative of the general population need to be studied to generalize the results. Additionally, due to the retrospective nature of the current study, we did not classify the included population according to clinical phenotypes of OA.

## 5. Conclusions

In conclusion, OA is increasing and becoming a worldwide health problem. Many biomarkers have been identified in numerous studies, mostly for scientific purposes. This study reported that blood SII was not a useful marker for predicting radiographic knee OA severity. It also supports the view that inflammatory mechanisms are involved in the pathogenesis of OA. Since SII is a simple and affordable biomarker, it is a simple and feasible method in usual clinical practice. The role of SII in evaluating OA severity should be further assessed in larger, prospective multicenter studies.

## Figures and Tables

**Table 1:** Demographic and laboratory data of patients.

	Group I *n* = 449 (%47.5)	Group II *n* = 497 (%52.5)	Total Study Population *n* = 946 (%100)	*p* Value
Age	54.00 (47.00–62.00)	66.00 (60.00–71.00)	61.00 (53.00–68.00)	<0.001
Gender				
Female	302 (%67.3)	398 (%80.1)	700 (%74.0)	<0.001
Male	147 (%32.7)	99 (%19.9)	246 (%26.0)	<0.001
WBC (×10^9^/L)	7.49 (6.31–8.82)	7.43 (6.28–8.71)	7.46 (6.29–8.73)	0.65
Hemoglobin (g/dL)	13.70 (12.90–15.00)	13.50 (12.60–14.60)	13.70 (12.70–14.70)	0.002
Lymphocyte (×10^9^/L)	2.36 (1.91–2.87)	2.07 (1.69–2.58)	2.20 (1.78–2.73)	<0.001
Neutrophil (×10^9^/L)	4.18 (3.35–5.25)	4.50 (3.56–5.68)	4.39 (3.46–5.50)	0.03
Platelet (×10^9^/L)	270.00 (234.00–314.00)	279.00 (233.50–319.50)	273.00 (234.00–318.00)	0.295
SII	474.54 (359.29–677.25)	578.14 (414.58–799.71)	536.72 (381.56–745.75)	<0.001

Comparisons of study population groups regarding age, gender, and biomarkers are presented. (WBC: White Blood Count; SII: Systemic Immune-inflammatory Index.)

**Table 2:** Relationship between systemic immune-inflammatory index (SII) and osteoarthritis (OA) severity.

	SII		*p* Value
	<627.9	>627.9	<0.001
Group I (Mild–Moderate OA)	317 (%52.6)	132 (%38.5)	
Group II (Severe OA)	286 (%47.4)	211 (%61.5)	
Total	603 (%100)	343 (%100)	946

(OA: Osteoarthritis; SII: Systemic Immune-inflammatory Index).

**Table 3:** Regression analysis results of markers affecting osteoarthritis (OA) severity.

	Univariate		Multivariate	
Parameter	OR (%95 CI)	*p* Value	OR (%95 CI)	*p* Value
Age	1.132 (1.112–1.153)	<0.001	1.131 (1.111–1.151)	<0.001
Hemoglobin	0.852 (0.786–0.924)	<0.001	0.920 (0.833–1.015)	0.096
SII	1.772 (1.353–2.321)	<0.001	1.623 (1.182–2.229)	0.003

(OR: Odds Ratio; CI: Confidence Interval; SII: Systemic Immune-inflammatory Index).

## Data Availability

Data is contained within the article.

## Abbreviations

The following abbreviations are used in this manuscript:

OA	Osteoarthritis
SII	Systemic Immune-Inflammatory Index
IL-1β	Interleukin-1β
TNF-α	Tumor necrosis factor-α
MMPs	Matrix Metalloproteinases
PGE2	Prostaglandin-E2
NO	Nitric Oxide
CRP	C-Reactive Protein
ESR	Erythrocyte Sedimentation Rate
IL-6	Interleukin-6
CBC	Complete Blood Count
WBC	White Blood Cell Count
NLR	Neutrophil-to-Lymphocyte Ratio
PLR	Platelet-to-Lymphocyte Ratio
MLR	Monocyte-to-Lymphocyte Ratio
PDW	Platelet Distribution Width
RDW	Red Cell Distribution Width
RPR	Rapid Plasma Reagin
MPV	Mean Platelet Volume
EDTA	Ethylenediaminetetraacetic Acid
Hgb	Hemoglobin
SLE	Systemic Lupus Erythematosus
RA	Rheumatoid Arthritis
BMD	Bone Mineral Density
AS	Ankylosing Spondylitis
POP	Postoperative Pneumonia
